# Supplementary figures and images for: Radiosensitizing Effect of Trabectedin on Human Soft Tissue Sarcoma Cells
Source: Int J Mol Sci. 2022 Nov 18;23(22):14305. doi: 10.3390/ijms232214305 (PMC9698158; doi:10.3390/ijms232214305)

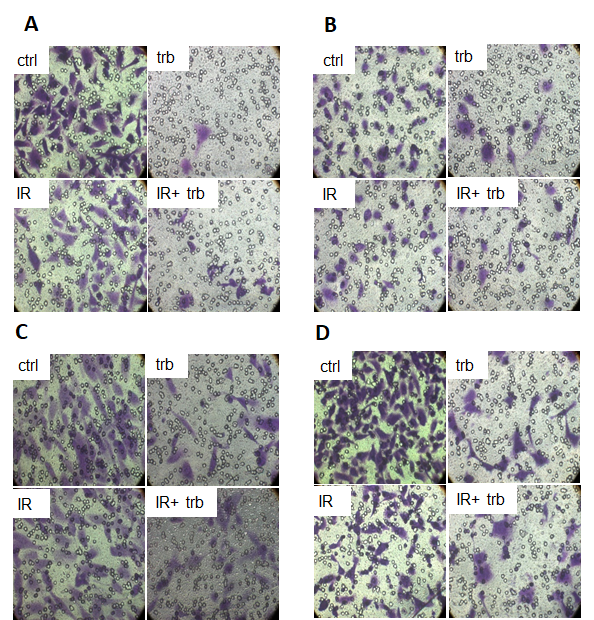

Supplement: Supplementary file 1 [file ijms-23-14305-s001.zip › Figure Supp1.tif]

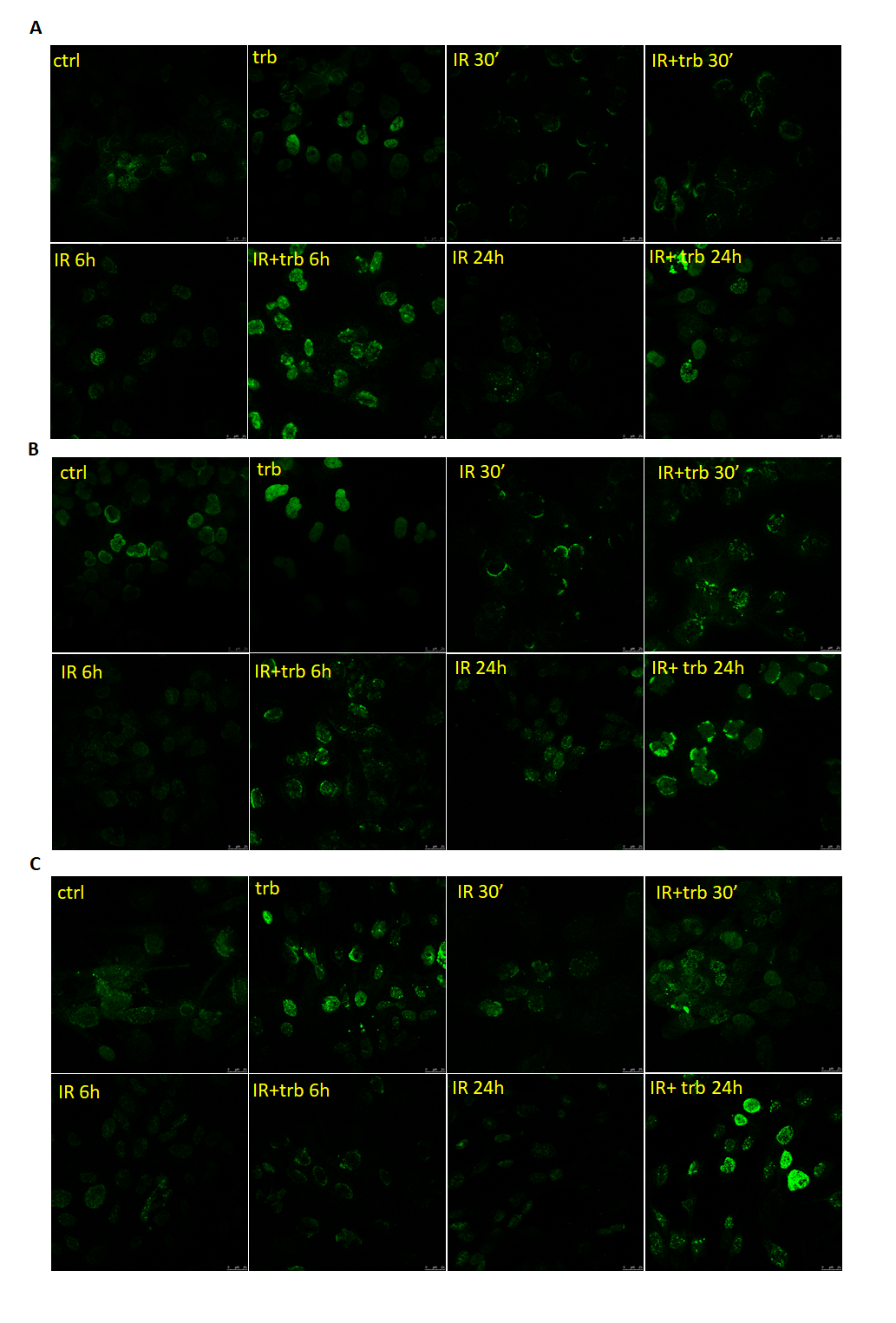

Supplement: Supplementary file 1 [file ijms-23-14305-s001.zip › Figure Supp2.tif]
